# Systemic and Mucosal Immune Reactivity upon *Mycobacterium avium* ssp. *paratuberculosis* Infection in Mice

**DOI:** 10.1371/journal.pone.0094624

**Published:** 2014-04-11

**Authors:** Arzu Koc, Imke Bargen, Abdulhadi Suwandi, Martin Roderfeld, Annette Tschuschner, Timo Rath, Gerald F. Gerlach, Mathias Hornef, Ralph Goethe, Siegfried Weiss, Elke Roeb

**Affiliations:** 1 Justus-Liebig-University Giessen, Department of Gastroenterology, Giessen, Germany; 2 Helmholtz Centre for Infection Research, Molecular Immunology, Braunschweig, Germany; 3 Medical Clinic 1, Friedrich-Alexander University Erlangen-Nuernberg, Erlangen, Germany; 4 IVD GmbH, Hannover, Germany; 5 Department of Microbiology, Hannover Medical School, Hannover, Germany; 6 Institute for Microbiology, Department of Infectious Diseases, University of Veterinary Medicine Hannover, Hannover, Germany; Inserm, France

## Abstract

Mycobacterium avium ssp. paratuberculosis (MAP) is the cause of Johne's disease, an inflammatory bowel disorder of ruminants. Due to the similar pathology, MAP was also suggested to cause Crohn's disease (CD). Despite of intensive research, this question is still not settled, possibly due to the lack of versatile mouse models. The aim of this study was to identify basic immunologic mechanisms in response to MAP infection. Immune compromised C57BL/6 *Rag2* ^−/−^ mice were infected with MAP intraperitoneally. Such chronically infected mice were then reconstituted with CD4^+^ and CD8^+^ T cells 28 days after infection. A systemic inflammatory response, detected as enlargement of the spleen and granuloma formation in the liver, was observed in mice infected and reconstituted with CD4^+^ T cells. Whereby inflammation in infected and CD4^+^CD45RB^hi^ T cell reconstituted animals was always higher than in the other groups. Reconstitution of infected animals with CD8^+^ T cells did not result in any inflammatory signs. Interestingly, various markers of inflammation were strongly up-regulated in the colon of infected mice reconstituted with CD4^+^CD45RB^lo/int^ T cells. We propose, the usual non-colitogenic CD4^+^CD45RB^lo/int^ T cells were converted into inflammatory T cells by the interaction with MAP. However, the power of such cells might be not sufficient for a fully established inflammatory response in the colon. Nevertheless, our model system appears to mirror aspects of an inflammatory bowel disease (IBD) like CD and Johne's diseases. Thus, it will provide an experimental platform on which further knowledge on IBD and the involvement of MAP in the induction of CD could be acquired.

## Introduction

Crohn's disease (CD) belongs to the family of human inflammatory bowel diseases and is believed to result from an excessive mucosal immune response towards the enteric microbiota in a genetically susceptible host [1]. Its histopathological characteristics are very similar to Johne's disease, a chronic granulomatous inflammation of the small intestine of ruminants that is caused by *Mycobacterium avium* ssp. *paratuberculosis* (MAP). Due to the histomorphological similarities MAP has already been suggested to be involved in the pathogenesis of CD in 1913 [2]. Indeed, a number of studies reported the detection of MAP in material obtained from CD patients [3]–[6]. However, other groups could not confirm these results and could detect MAP in a significant number of apparently healthy individuals as well [7]. A possible causative role of MAP for CD is therefore still under debate.

MAP is one of the mycobacteria that exhibits a very long generation time. Similar to other species of this genus, MAP is able to survive even under harsh environmental conditions for long periods of time [8], [9]. Infections are mainly observed in ruminants although sporadic infections of primates and many other species have been described [10]. In most instances, transmission occurs during the neonatal/infant period via the oral-faecal route and M cells are believed to represent the main mechanism of mucosal translocation followed by phagocytosis in subepithelial macrophages [11], [12].

Similar to other pathogenic mycobacterial species, MAP is able to survive and proliferate in the phagosome. Infected macrophages might further function as “trojan horse” and facilitate dissemination of MAP to other tissues [11], [13], [14]. Animals develop clinical signs of infection as recently as months to years after infection. Weight loss, reduced lactation and chronic diarrhea associated with wasting and shedding of large numbers of bacteria are observed [15]. Histopathological analysis reveals severe intestinal mucosal inflammation and granulomas in the small and large intestine as well as the liver [16].

The delayed onset of disease after an extended period of latency led us to hypothesize that MAP might initially be controlled by the host's immune system. After impairment of immune function by stress or additional infection, proliferation of MAP might cause clinically apparent infection and fatal outcome. The well-described reactivity of both the innate as well as the adaptive immune system towards mycobacteria might then drive the inflammatory symptoms observed during manifest disease.

In order to simulate such a situation, we selected immune compromised mice that lack the recombination activating genes (*Rag^−/−^*). These genes are required for the rearrangement of the gene segments forming the T and B cell receptors. As consequence, such mice lack cells of the adaptive immune system and are severely immune compromised [17]. Infection by intraperitoneal injection was selected since no *in vivo* mouse model of mucosal translocation has been reported. Infected mice were subsequently reconstituted with various T cell subpopulations and liver and intestinal tissues were screened for signs of immune activation such as granuloma formation or up-regulation of matrix metalloproteinases (MMPs), tissue inhibitors of metalloproteinases (TIMPs), Toll like receptors (TLR), and pro-inflammatory cytokines. MMPs are the most potent proteases in the turnover of the extracellular matrix [18] but also known to either stimulate or maintain inflammation by proteolytic processing of inflammatory cytokines [19]. Importantly, MMPs are known to play a critical role in mucosal barrier function and inflammatory bowel disease (IBD) [20], [21].

CD45RB is a member of protein tyrosine phosphatase family expressed on leukocytes and known as an essential regulator in T lymphocytes [22]. Adoptive transfer of CD4^+^CD45RB^hi^ T cells (naive T cells) from healthy wild-type to lymphopenic mice leads to colitis and small bowel inflammation at 5–8 weeks following T cell transfer which represents an important model to study specific T cells involvement in dysregulation [23]. Coinjection of CD4^+^CD45RB^lo^ cells (activated/memory T cells) could prevent the development of colitis. It was shown that the CD4^+^CD45RB^lo^ population contains CD25^+^Foxp3^+^ regulatory T cells (Treg cells), which are responsible for the regulatory activity of this T cells subset [24].

By T-cell reconstitution of MAP-infected immune compromised *Rag2^−/−^* mice we now show for the first time that MAP-induced systemic inflammation is mainly driven by CD4^+^CD45RB^hi^ T cells. Under the influence of MAP CD4^+^CD45RB^lo/int^ T cells convert into effector cells during enteric mucosal inflammation.

## Materials and Methods

### Bacterial culture

The MAP strain DSM 44135 was cultured and prepared for infection as previously described [25]. For infection experiments, bacteria were transferred to Dulbecco's Modified Eagle Medium and bacterial suspensions were vortexed in the presence of glass beads (3 mm diameter) for 15 min, centrifuged for 10 min at 2900 g and washed with PBS. Infection doses were calculated by determining the optical density measured at 600 nm of the supernatants containing single bacteria. An OD_600_ of 0.1 corresponds to 10^7^ MAP/ml [26].

### Animals

C57BL/6 *Rag2^−/−^* mice were bred at the animal facility of the Helmholtz Centre for Infection Research (HZI) and maintained under specific pathogen-free conditions. Wild type (WT) C57BL/6 mice were purchased from Janvier (France). All animal experiments were done at HZI using adult mice between 8 and 12 weeks of age. Intraperitoneal (i.p.) infection was done with 10^8^ MAP in 200 µl PBS. Control mice were always inoculated with the same amount of PBS, respectively. This study was carried out in strict accordance with the German Law for the Protection of Animals. The protocol was approved by the Lower Saxony authorities (anim. exp. no. 33.11.42502-04-090/08, Niedersächsisches Landesamt für Verbraucherschutz und Lebensmittelsicherheit).

### Adoptive transfer experiments

Two groups of 4 mice each were inoculated i.p. with 10^8^ CFU MAP in 200 µl PBS. Additionally, 2 groups of 4 mice each were inoculated i.p. with PBS as control. 28 days later one group of infected and one group of uninfected mice were reconstituted by adoptive transfer. Spleen cells from naive C57BL/6 wild type mice were used for reconstitution of C57BL/6 *Rag2^−/−^* mice. In all groups recipient and donor mice were gender-matched. Murine spleens were flushed on ice with IMDM (Gibco BRL, Eggenstein, Germany) supplemented with 10% heat inactivated FCS and 0.25 mM β-mercaptoethanol. Red blood cells were lysed for 2 min in ACK lysis buffer (0.15 M NH_4_Cl, 10 mM KHCO_3_, 0.1 mM Na_2_EDTA in ddH_2_O) and B cells were removed using 25 µl/ml magnetic B cell removal beads (Invitrogen Dynabeads Mouse pan B (B220) 114.41D 4×10^8^ beads/ml). Cells were then incubated for 7′ with 500 µl 1∶500 diluted mouse serum (Biowest S2160-020) to block Fc receptors. The suspension was diluted to 14 ml with PBS and centrifuged 7′ at 1000 rpm (209×g). The pellet was resolved in 3 ml PBS and FcBlock (rat anti-mouse CD16/CD32 BD Pharmingen #5531422.4G2 0.01 µg/ml) and incubated for 7′ on ice. Cells were mixed with an equal volume of antibody solutions (1∶1) and incubated for 15′ in the dark. Cells were then washed and incubated with PI (Sigma P4170, 0.5 µg/ml) or DAPI (Sigma 9564, 10 µg/ml) for live/dead discrimination. For sorting cells were gated on live cells leaving out doublets. Dependent on the set up the following cell populations were sorted using a FACSAriaII cell sorter (Becton Dickinson, NJ, USA. using FACSDiva software): CD3^+^CD19^−^CD11c^−^CD4^+^CD45RB^lo/int^ (CD4^+^CD45RB^lo/int^ T cells), CD3^+^CD19^−^CD11c^−^CD4^+^CD45RB^hi^ (CD4^+^CD45RB^hi^ T cells), CD3^+^CD19^−^CD11c^−^CD8^+^ (CD8^+^ T cells). For staining the following antibodies were used: hamster anti mouse CD3e 145-2C11 PE (BD Pharmingen 20 µg/ml), rat anti mouse CD19 1D3 APC (BD Pharmingen 2 µg/ml), hamster anti mouse CD11c N418 PECy7 (eBioscience 6.7 µg/ml), rat anti mouse CD4 RM4-5 APCeFlour780 (eBioscience 10 µg/ml), rat anti mouse CD45RB 16A FITC (BD Pharmingen 1.7 µg/ml), rat anti mouse CD8a 53-6.7 PacificBlue (eBioscience 10 µg/ml). After sorting, the cells were counted and the cell number was adjusted to 2×10^6^ cells in 150 µl PBS and injected i.v. Body weight of the mice was monitored regularly as read out for the general health of the animals. Four weeks after adoptive transfer the mice were sacrificed and organs were removed for further analysis.

### Liver plating

Liver were homogenized with sterile PBS in the presence of sterile 3 mm glass beads 2 times for 20 seconds using the homogenizer FastPrep-24 (MP Biomedicals). The liver homogenates were plated on Middlebrook 7H10 agar (Difco TM) containing Mycobactin J (IDVet Innovative Technology). The plates were incubated at 37°C for up to 4 weeks. After this incubation time the plates were analyzed. Every single white dot was defined as one colony forming unit (CFU). The CFU of the whole plate were counted and the CFU/g liver was calculated according the dilution factor.

### DNA preparation with Qiagen QIAmp DNA Stool kit

300 mg liver was homogenized with 1.4 ml buffer ASL and 0.1 mm, 1.4 mm and 3 mm glass beads 6 times 40″ with the MP FastPrep-24. After centrifugation for 1′ at 13000 rpm (16089×g), the solution was incubated 20′ at 95°C. For further steps Qiagen QIAmp DNA Stool kit was used due to manufacturers' instructions. The DNA was used for PCR as a 1∶100 dilution in ddH_2_O.

### Polymerase chain reaction

Polymerase chain reaction (PCR) was done with Fermentas True Start Hot Start Taq DNA polymerase. Mastermix for one reaction was prepared as follows: 7.7 µl water (Ampuwa Fresenius Kabi), 1.6 µl MgCl_2_, 2 µl buffer, 1 µl forward primer (5 pmol/µl), 1 µl reverse primer (5 pmol/µl), 1.6 µl dNTPs (10 mM dNTP mix Bioline), 0.1 µl Taq polymerase. 15 µl mastermix were mixed with 5 µl DNA. The PCR program was as follows: 3′ at 95°C 35 cycles of 30″ 95°C, 30″ 61°C and 30″ 72°C followed by 3′ 72°C and 5′ 8°C (peqLab Biotechnologie GmbH peqStar).

Primer: MAP-IS900-F 5′-AATGACGGTTACGGAGGTGGT-3′ and MAP-IS900-R 5′-GCTGCGCGTCGTCGTTAATA-3′

### RNA extraction and mRNA expression

Shock-frozen tissue samples (10–20 mg) from the colon (middle part) and small intestine (end part) were homogenized with Precellys Ceramic Beads, 1.4 mm (Peqlab, Erlangen, Germany) and total cellular RNA was extracted using Qiagen RNeasy Mini Kit reagent (Qiagen, Hilden, Germany) according to the manufacturer's protocol. First strand cDNA synthesis was performed using iScript reverse transcriptase (Bio-Rad Laboratories CA), according to the manufacturer's instructions. qRT-PCR was performed in duplicates using a Mx3005P Cycler (Stratagene, La Jolla, CA). Primers were purchased from Qiagen, Hilden, Germany (Table S1). The specificity and sensitivity of the qPCR was confirmed by analysis of molecular weight and melting points of the products. The expression of the gene of interest was normalized against r18S mRNA. All data were analyzed by the ΔΔCt model [27], [28].

### Immunohistological analysis

Immunohistological stainings of colon samples were performed as described [29].

### Statistical analysis

Statistical analyses were performed with SPSS 19.0 software (SPSS Inc. Chicago, Illinois, USA). Considering not normally distributed parameters non-parametric tests were applied. The data were analysed using Mann-Whitney U-test. Results are presented as mean ± standard error of mean (SEM). A two-sided *p*<0.05 was considered significant.

## Results

### MAP Infection and T cell reconstitution of *Rag2^−/−^* mice

**Figure 1:**
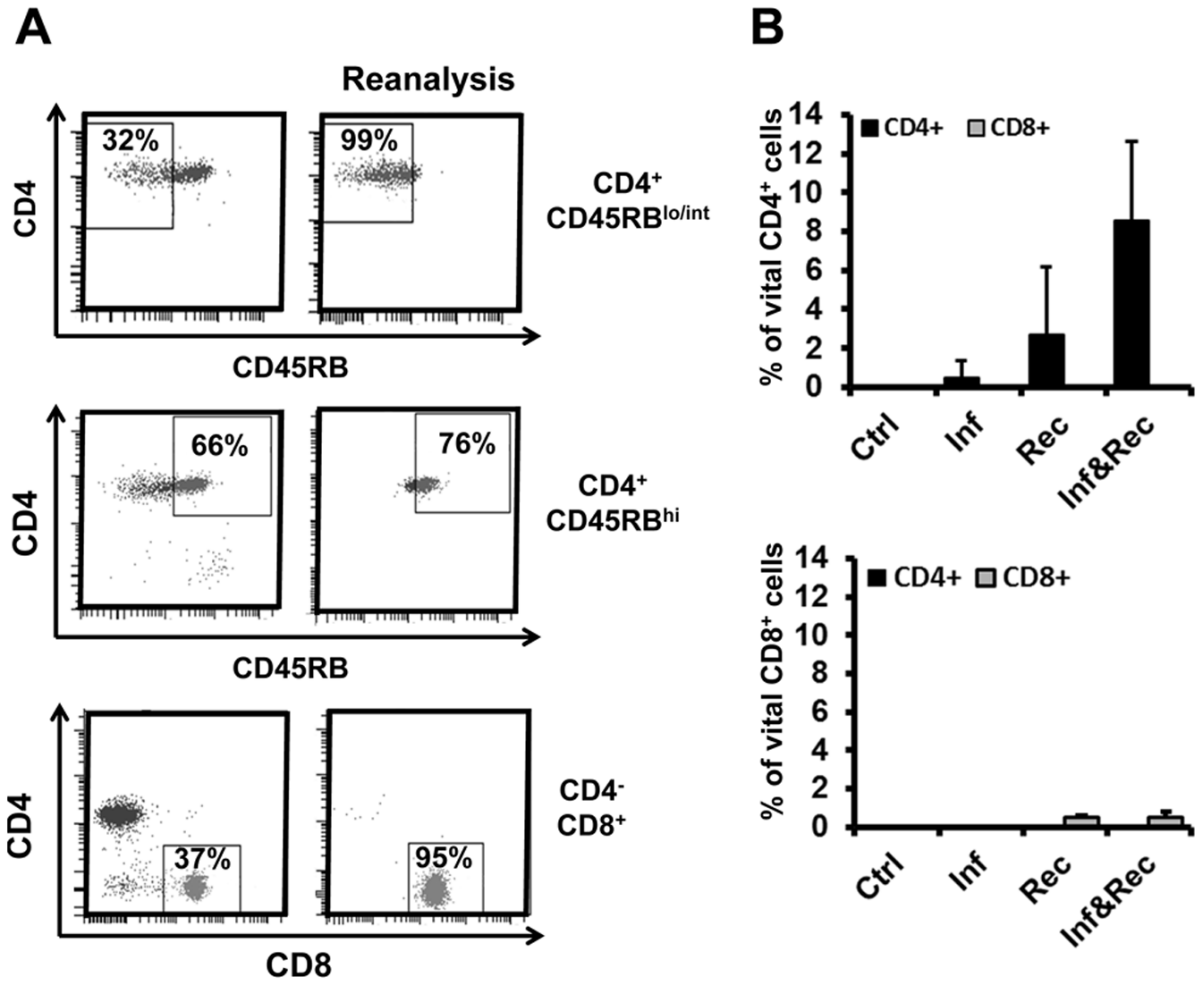
Adoptive transfer of T cell populations into MAP infected *Rag2* ^−/−^ mice. **A.** Sorting strategy and reanalysis of CD4^+^CD45RB^lo/int^, CD4^+^CD45RB^hi^ and CD8^+^ T cells from spleen of naïve mice. Gates were set to sort pure population of CD3^+^CD19^−^CD4^+^CD45RB^lo/int^, CD19^−^CD3^+^CD8^−^CD4^+^CD45RB^hi^, CD3^+^CD19^−^CD4^−^CD8^+^ T cells. **B**. Analysis of reconstituted mice for CD4^+^ and CD8^+^ T cells four weeks after transfer i.e. day 56 of the experiment. Ctr – control; Inf – only infected with MAP at the beginning of the experiment, Rec – reconstituted with the T cell population indicated; Inf & Rec – infected at the beginning of the experiment and reconstituted after 4 weeks.

The experiments were outlined to simulate a situation in which MAP infected mice recover from cellular immune hyporesponsiveness. Immunocompromised *Rag2^−/−^* mice were left untreated or infected i.p. with 10^8^ CFU MAP. After four weeks, animals were reconstituted with purified total spleen cells (data not shown), CD4^+^ or CD8^+^ T cell subpopulations (Figure 1). The CD4^+^ T cells were further separated according the expression levels of CD45RB. Whereas CD45RB^hi^ have previously been shown to promote mucosal inflammation in immune compromised mice, this phenotype is not observed after transfer of CD45RB^lo/int^ cells [23].

**Figure 2:**
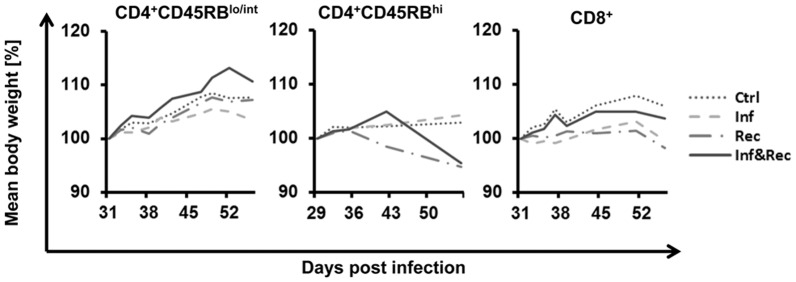
Body weight of *Rag2^−/−^* mice after MAP infection and/or T cell reconstitution. Infection with MAP led to hardly any decrease of body weight even not in the first week. Adoptive transfer of CD4^+^CD45RB^hi^, CD4^+^CD45RB^lo/int^ or CD8^+^ T cells on day 28 had no influence on the body weight, neither of uninfected nor of infected mice. n = 4 mice in each group.

Purity of the cell populations was verified prior to reconstitution by FACS re-analysis (Figure 1A). In addition, reconstituted mice were assessed for splenic CD4^+^ and CD8^+^ T cells. No CD8^+^ T cells were detected in 4 of 4 mice reconstituted with CD4^+^ T cells. Vice versa, in 4 of 4 mice reconstituted with CD8^+^ T-cells, no CD4^+^ T cells were found, thereby verifying the specificity of the reconstitutions (Figure 1B). Interestingly, the percentage of vital CD4^+^ T cells in reconstituted mice infected with MAP is higher compared with controls. Possibly MAP infection led to activation of such T cells. As a general indicator of health status, no changes in the body weight were observed after infection and/or reconstitution (Figure 2).

### Signs of systemic inflammation in reconstituted MAP infected *Rag2^−/−^* mice

**Figure 3:**
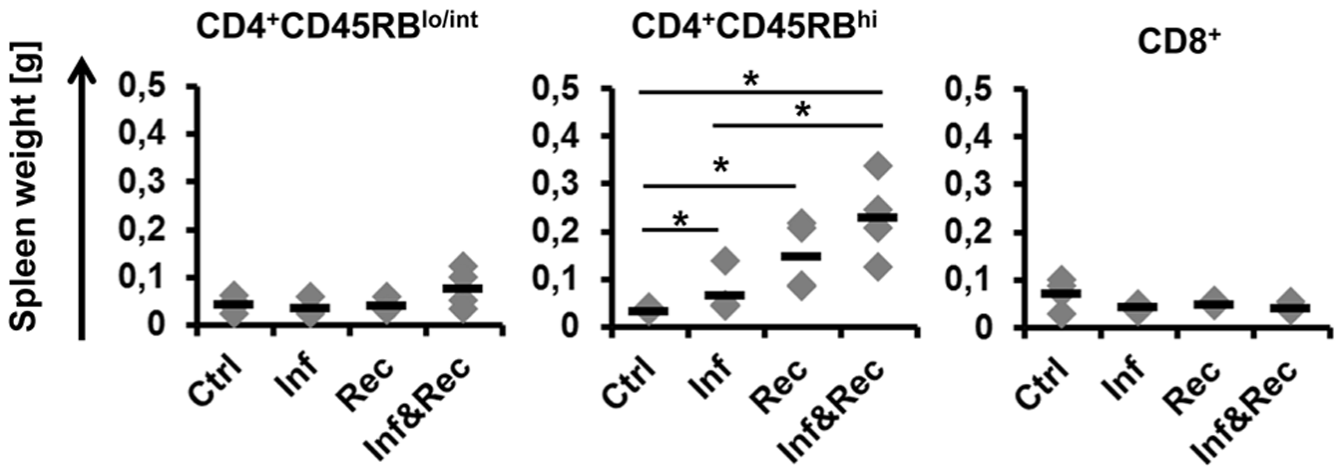
Increase of spleen weight in MAP infected and CD4^+^ T cell reconstituted *Rag2^−/−^* mice 8 weeks. Three different T cell subsets were used for adoptive transfer 4 weeks post infection: CD4^+^CD45RB^hi^, CD4^+^CD45RB^lo/int^ and CD8^+^. Each group with n = 4 mice. Mann-Whitney U-Test p = 0.05*, p = 0.01**. The experiment was carried out at least twice with similar results.

In addition, total organ weight of spleen tissue was assessed. Interestingly, spleens from 3 of 4 infected mice reconstituted with colitogenic CD4^+^CD45RB^hi^ T cells exhibited markedly enhanced weight (Figure 3). In contrast, reconstitution of infected *Rag2^−/−^* mice with CD4^+^CD45RB^lo/int^ or CD8^+^ T cells did not result in enhanced spleen weight in 2 of 4 mice (Figure 3).

MAP is known to induce liver granulomas in cattle. Similarly, granuloma formation has been observed in MAP infected wild type mice (data to be published). Therefore, liver tissues from all groups were formalin fixed and stained with haematoxylin/eosin. As expected no granulomas were observed in control *Rag2^−/−^* mice and non-reconstituted *Rag2^−/−^* mice infected with MAP (Figure S1). This finding is in accordance with the critical role of the adaptive immune system (and in particular the critical role of certain T cells) for the generation of granulomas. Also, no granulomas were observed in mice reconstituted with CD8^+^ T cells consistent with the previous finding that CD8^+^ T cells are not involved in granuloma formation [30], [31].

**Figure 4:**
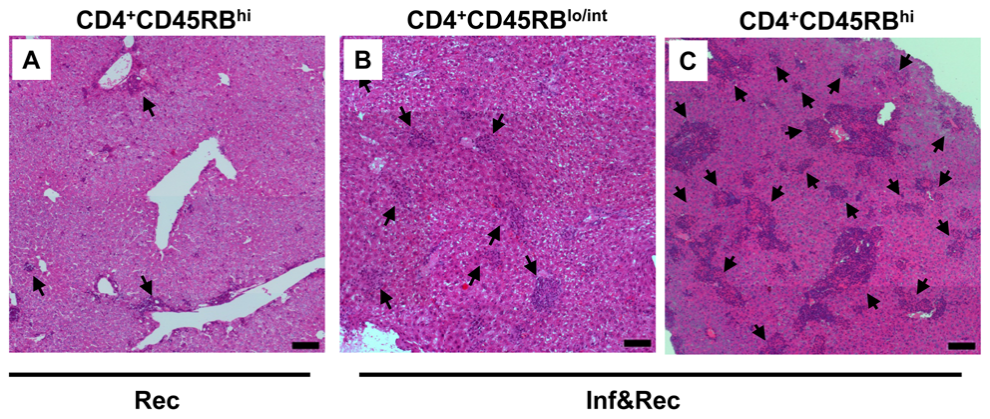
Histology of liver from T cell reconstituted *Rag2^−/−^* mice infected with MAP or not. **A.** MAP infected and CD4^+^CD45RB^lo/int^ T cell reconstituted (Inf&Rec) *Rag2^−^* ^/*−*^ mice. **B**. Control of *Rag2^−/−^* mice reconstituted with CD4^+^CD45RB^hi^ T cells not infected with MAP. **C**. *Rag2^−/−^* mice infected with MAP and reconstituted with CD4^+^CD45RB^hi^ T cells. Controls are shown in Fig. S1. Arrow heads point at the granulomatous structures. Adoptive transfer of CD8^+^ T cells after MAP infection did not lead to formation of granulomatous structures. Bars depict 100 µm. The data are representative from 4 mice per group and the experiments were carried out at least twice with similar results.

Surprisingly, liver granulomas were found in 1 of 4 MAP infected mice reconstituted with CD4^+^CD45RB^lo/int^ T lymphocytes but not in their uninfected counterparts (Figure 4 and Figure S1). In contrast, granulomas were observed in liver tissue of mice reconstituted with the colitogenic subpopulation of CD4^+^CD45RB^hi^ T cells even in the absence of MAP infection. However, in MAP infected animals reconstituted with CD4^+^CD45RB^hi^ T cells more and significantly larger granulomas were found in all 4 mice compared to mice that received CD4^+^CD45RB^hi^ T cell reconstitution but no MAP infection (Figure 4 and Figure S1).

**Figure 5:**
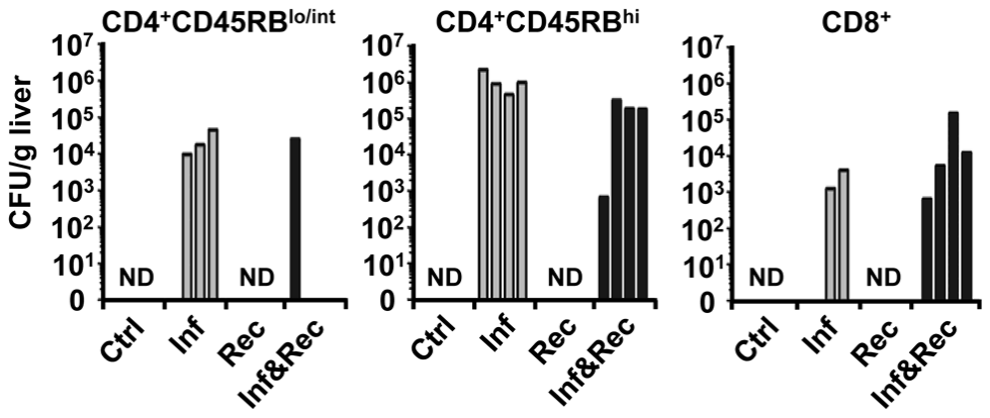
MAP colony forming units derived from liver tissue of *Rag2^−/−^* mice 8 weeks post infection. Three different T cell subsets were used for adoptive transfer 4 weeks post infection: CD4^+^CD45RB^hi^, CD4^+^CD45RB^lo/int^ and CD8^+^. Only infected as well as infected and reconstituted animals contained colony forming units i.e. MAP, as expected. Although all mice of a group were injected i.p. with 10^8^ MAP, plating 8 weeks later did not indicate a homogeneous infection. From some infected mice viable MAP could not be revealed from liver. Similar data were obtained using PCR. Each group n = 4 mice. The experiments were carried out twice with similar results.

Thereby, it is apparent that MAP infection is either an absolute prerequisite for the development of T cell mediated granuloma formation (as seen for CD4^+^CD45RB^lo/int^ T cells) or a strongly promoting factor in this process (as seen for CD4^+^CD45RB^hi^ T cells). In addition, granulomas were only found in liver tissue in which MAP could be detected. Although MAP was only occasionally detected by Ziehl-Neelsen staining in liver sections of infected mice, the presence of low numbers of viable MAP was confirmed either by serial dilution and plating of liver homogenates (Figure 5) or alternatively by PCR (for results please refer to Table S2).

### Colonic inflammatory response by CD4^+^CD45RB^lo/int^ T cells in MAP infected mice

CD4^+^ T cells of the CD45RB^hi^ type are known to induce colitis in lymphopenic mice most likely due to the absence of regulatory T cells in this lymphocyte population. We therefore concentrated on the question whether CD4^+^ T cells of the CD45RB^lo/int^ type would be converted into inflammatory T cells by the interaction with MAP. We hypothesized that, in the presence of MAP, CD4^+^CD45RB^lo/in^ T cells could produce inflammatory factors by themselves or stimulate other cells to produce them, and thus be converted into a pro-inflammatory phenotype.

**Figure 6:**
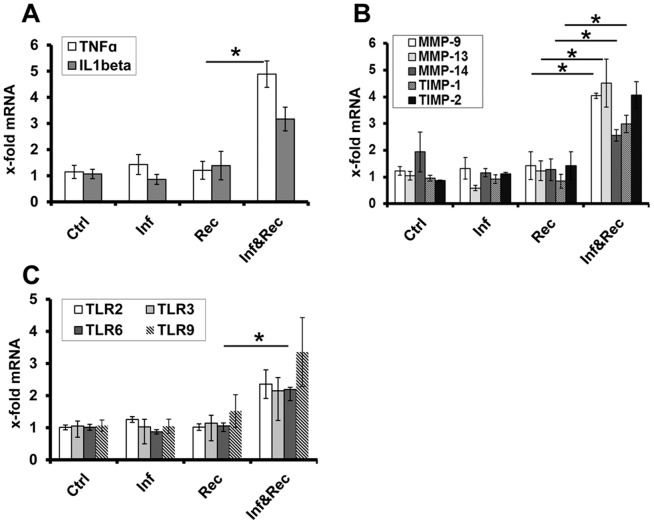
Colonic inflammatory response reconstitution of MAP infected *Rag2^−/−^* mice with CD4^+^CD45RB^lo/int^. **A.** Colonic expression of TNF-α and IL-1β. **B**. Colonic expression of MMP-9, MMP-13, MMP-14, TIMP-1 and TIMP-2. **C**. Colonic expression of TLRs. Mice had been infected i.p. with 10^8^ CFU MAP and 4 weeks later reconstituted with CD4^+^CD45RB^lo/int^ T-cells. Reconstituted and MAP infected mice compared to mice with reconstitution only. No differences in expression of the indicated genes were found in samples without T-cell reconstitution (data not shown). The expression of the genes of interest was normalized against r18S RNA. Ctrl =  *Rag2^−/−^* mice treated with PBS only; Inf  =  *Rag2^−/−^* mice infected with MAP; Rec  =  *Rag2^−/−^* mice reconstituted with CD4^+^CD45RB^lo/int^; Inf&Rec  =  *Rag2^−/−^* mice infected with MAP and reconstituted with CD4^+^CD45RB^lo/int^. n = 4. Bars depict median ± SEM. Statistical significance (p<0.05) is indicated by *.

First, we analyzed tumor necrosis factor-α (*TNF-α*) and interleukin 1β (IL-1β). *TNF-α* is a potent pro-inflammatory cytokine with elevated levels found in several autoimmune diseases including rheumatoid arthritis and CD [32]–[34]. Consistent with our hypothesis, transcriptional levels of *TNF-α* were significantly increased (4.9-fold, p = 0.049) in the colon of mice with MAP infection and CD4^+^CD45RB^lo/int^ T-cell reconstitution, but not mice with MAP infection only (without concomitant T cell reconstitution) or those with CD4^+^CD45RB^lo/int^ reconstitution only. *IL-1β* expression was higher by trend in mice with MAP infection and CD4^+^CD45RB^lo/int^ T-cell reconstitution (Figure 6A).

Having shown an increase of the pro-inflammatory cytokine *TNF-α* in the presence of MAP and CD4^+^CD45RB^lo/int^ T cells, we turned our attention to factors responsible for maintaining inflammation and tissue destruction and therefore determined the expression levels of *MMPs* as well as their inhibitory regulators, the *TIMPs* [35], [36].

As demonstrated in Figure 6B, a significant increased expression of *MMP-9* (4-fold, p = 0.043), *MMP-13* (4.5-fold, p = 0.021), *MMP-14* (2.6-fold, p = 0.043) and *TIMP-1* (3-fold, p = 0.021) was observed in MAP-infected *Rag2^−/−^* mice reconstituted with CD4^+^CD45RB^lo/int^ T cells. Similarly to the results seen for *TNF-α*, these markers remained unaltered in their expression level in *Rag2^−/−^* mice infected with MAP only or those reconstituted with CD4^+^CD45RB^lo/int^ T cells only. Thus, strengthening our hypothesis that both, MAP and CD4^+^CD45RB^lo/int^ T cells, and possibly their interaction, are critical for the induction and maintenance of inflammation in the model used.

TLRs are critically involved in the recognition of microbe-associated molecular patterns and in the initiation of an innate immune response upon bacterial challenge. Therefore, we further quantified the expression of TLRs involved in the recognition of mycobacterial components. Consistent with its prominent role for the recognition of MAP [29], [37], [38], we found a significant upregulation of *TLR-6* (2.2-fold, p = 0.043) in mice that received both, MAP infection and concomitant CD4^+^CD45RB^lo/int^ T-cell reconstitution compared to mice with MAP infection or T cell reconstitution only. *TLR-2, -3*, and *-9* showed similar trends of expression in mice with MAP infection and CD4^+^CD45RB^lo/int^ T-cell reconstitution, although did not reach statistical significance (Figure 6C).

### Immunohistochemistry of MAP infected CD4^+^CD45RB^lo/int^ reconstituted *Rag2^−/−^* mice

**Figure 7:**
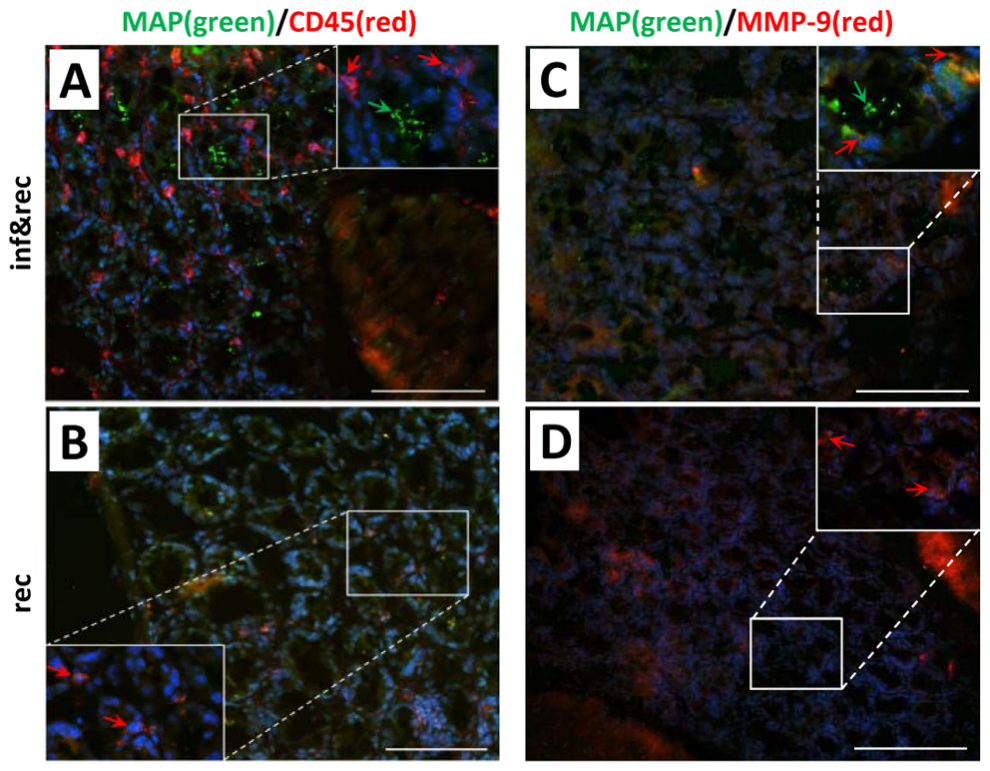
Immunofluorescence staining of murine colon from MAP infected *Rag2^−/−^* mice reconstituted with CD4^+^CD45RB^lo/int^ T-cells and in non-infected but reconstituted mice for control. **A–B**. Samples were stained for MAP (green) and leukocytes (CD45: red). Green arrows indicate colonies of MAP in the epithelium and red arrows indicate leukocytes most likely macrophages. **C–D**. Samples were stained for MAP (green) and MMP-9 (red). Green arrows indicate colonies of intracellular MAP. Red arrows indicate MMP-9 expressing leukocytes. In reconstituted and non-infected mice no MAP clusters could be detected and showed a normal histology of colon mucosa. Magnification 200x&1000x, bar = 100 µm. Blue staining in all samples: DAPI. The data are representative from 4 mice per group and the experiment was carried out at least twice with similar results.

Despite the fact that the peritoneum was chosen as the site of MAP infection in the current study, MAP was occasionally detected in colonic tissue of all 4 MAP infected mice (reconstituted with CD4^+^CD45RB^lo/int^) using a polyclonal antiserum against the protein MAP 1775 (Figure 7). In contrast, no staining was detected in non-infected animals, thereby confirming that these results were indeed due to the i.p. infection with MAP, but not due to an environmental presence of MAP. These results were further corroborated using a highly MAP-specific PCR, by which MAP transcripts were detected in the colon of all mice, that received MAP infections but not in non-infected controls. Interestingly, immunofluorescence staining demonstrated intracellular clusters of MAP associated with and surrounded by aggregates of CD45^+^ leukocytes (Figure 7A). Further, *MMP-9* expressing cells could be demonstrated in close proximity or directly adjacent to MAP clusters (Figure 7B).

## Discussion

Based on pathological similarities between human CD and Johne's disease in ruminants MAP was suggested as causative agent of CD almost 100 year ago. However, epidemiological and clinical studies have since then failed to provide unambiguous evidence for a causative role of MAP in the pathogenesis of CD [15]. Geographical variation in MAP exposure, major technical difficulties to culture MAP from primary tissue samples and multiple variants of this complex disease entity might, however, have blurred the picture. In the past, the establishment of animal models and the use of defined, genetically homogenous groups of individuals facilitated deeper insights into fundamental questions of mucosal inflammation and provided clearly defined hypotheses to be subsequently investigated in humans. Unfortunately, no mouse or small animal model has been reported to date that would allow experimental approaches on the association of MAP with CD. One might argue that this, among other factors, might be one of the reasons why the potential role of MAP in the pathogenesis of CD still has remained unresolved.

In the present work we aimed to mimic the situation that might underlie the pathogenesis of CD and Johne's disease. We considered that MAP infection in ruminants takes place during the neonatal period, a transient period of immunosuppression, leading to manifest clinical disease in later years of life. Based on these considerations, we injected MAP i.p. into immunocompromised *Rag2^−/−^* mice that lack B and T cells and by complementation with defined lymphocyte subpopulations subsequent to MAP infection. This approach allowed the identification of the cellular immune function responsible for MAP-induced inflammation. Although the way of infection utilized by us does not represent the physiological route of MAP infection, it consistently resulted in chronic infection within the current study. In this context the following is important to note: in contrast to i.p. injection, oral infection of adult mice with MAP does not allow reliable infection and thus is not suitable to study MAP host interaction [39]. Further, as shown within this study, i.p. infection leads to systemic spread of MAP, infection of liver tissue and significant infection-induced immunological alterations in the colon, thereby illustrating its potential value for the analysis of MAP-induced inflammation.

Using this approach, we are able to demonstrate that systemic inflammation, granuloma formation and intestinal expression of *TNF-α* was linked to CD4^+^ T cell populations whereas, as expected, CD8^+^ T cells did not induce MAP mediated pathology. To be more precise, granuloma formation mediated by CD4^+^CD45RB^lo/int^ T cells was exclusively detected in MAP infected mice whereas transfer of CD45RB^hi^ cells induced granuloma formation even in the absence of MAP infection, illustrating the potent inflammatory potential of this cell population, as reported before [16]. Nevertheless, granulomas were clearly increased in numbers and size in mice with both, MAP infection and reconstitution of CD4^+^CD45RB^hi^ T cells, indicating that MAP infection significantly increases the potential of distinct T cell subpopulations to attract inflammatory cells like macrophages and neutrophils to the site of infection.

As CD4^+^CD45RB^hi^ T cells apparently exhibited pro-inflammatory properties per se [40], we focused on the effect of CD4^+^CD45RB^lo/int^ T cells in subsequent analyses. Several markers of inflammation and tissue regeneration and MAP recognition were quantified.

We started off by analyzing expression levels of *TNF-α* and *IL-1β* as broad and potent pro-inflammatory cytokines and the utilized experimental strategy that allowed us to dissect the effects of combined MAP infection and CD4^+^CD45RB^lo/int^ T cell reconstitution, and of both components (MAP and CD4^+^CD45RB^lo/int^ T cells) alone on expression of these cytokines. Using this approach, we were able to show that MAP infection and subsequent reconstitution of the CD4^+^CD45RB^lo/int^ T cell population led to strong induction of *TNF-α* (results for *IL-1β* were not significant). Of note, this increase of *TNF-α* was not observed in mice with MAP infection alone or CD4^+^CD45RB^lo/int^ T cell reconstitution alone, we take this as evidence that MAP converts the reconstituted CD4^+^CD45RB^lo/int^ T cells into a pro-inflammatory phenotype. These findings are consistent with data in the literature: elevated *TNF-α* level have been found in several diseases with autoimmune components including rheumatoid arthritis and CD and importantly, in the context of MAP infection, *in vitro* studies demonstrated *TNF-α* secretion by mucosal organ cultures obtained from MAP positive CD patients [32], [34].

Based on previous results from our group [41], [42] we further chose to quantify *MMPs* and *TIMP-1* which are key enzymes in the matrix turnover and tissue destruction in inflammatory bowel diseases and also indicate pathogenic relevance during mycobacterial infections [43]. In this regard, previous studies have identified *MMP-9* as a key enzyme during mycobacterial infections [44], [45]. Consistent with these data, the observed enhanced expression of *MMP-9* in MAP infected and CD4^+^CD45RB^lo/int^ T cell reconstituted mice, but not in mice with MAP infection or T cell reconstitution alone, could represent a part of the host immune response towards MAP and might disclose an essential role in mediating mycobacterial pathogenicity [29], [46]. Apart from *MMP-9*, we observed an induction of *MMP-13* and *MMP-14* in MAP infected and T cell reconstituted mice. Nevertheless, as we could not observe any tissue destruction by histological examination, the role of induced MMP-expression in the interplay with CD4^+^CD45RB^lo/int^ T cells is still incomprehensible.

As TLRs play a key role in the recognition of microbe associated molecular patterns and the initiation of an innate immune response towards infectious agents, we further quantified members of the TLR family. Especially *TLR-2* and *TLR-6* have been shown to play an important role in the innate immune response against mycobacterial infections [47]–[49]. Consistent with reported data on the role of *TLR-6* for the recognition of MAP, we observed a significantly increased expression of *TLR-6* after MAP infection in CD4^+^CD45RB^lo/int^ T cells reconstituted *Rag2^−/−^* mice.

In summary and consistent with our hypothesis that both, MAP and T cells are required for the induction of inflammation and tissue pathology, we herein describe the induction of *TNF-α* and tissue destructive proteases only in the presence of MAP and CD4^+^CD45RB^lo/int^ T cells, but not under conditions where only one of the later factors is present. This might led to the hypothesis that MAP is, upon its recognition by the host, capable to initiate a host response that induces a pro-inflammatory and tissue destructive environment that subsequently might lead to manifest inflammation.

However, within this study, microscopic inflammation was restricted to the colon and despite the presence of inflammatory cells and inflammatory effector molecules, neither macroscopic colonic inflammation was detected nor histologic alterations were observed. It might be argued that the observation period after T cell reconstitution was not long enough for the development of macroscopic inflammation. Also, it is conceivable that a further “hit” such as genetic predisposition is necessary for the manifestation of full macroscopic colitis and that MAP and induction of cytokines by MAP represent a potent trigger in this setting. Clearly, these issues have to be addressed in the future and the approach presented here may provide a suitable animal model.

Further, MAP was not always detected in colonic tissue by immunohistochemistry and PCR. Thus, even in the absence of high bacterial burden, MAP infection prompted transferred CD4^+^ T cells particularly to inflammation and the formation of granulomas in liver tissue. This finding may be interesting in the context of the role of MAP in CD patients that show great variability in the detection rate of MAP in a number of recent studies.

In conclusion, we present an infection model that allows the analysis of the MAP induced stimulation and pro-inflammatory activity of CD4^+^ T cells. Following systemic infection, we observed significant signs of systemic infection, granuloma formation within the liver and inflammatory reactions in the colon. In addition viable MAP was cultured from inflamed tissue and the MAP-induced inflammatory potential of T cell subpopulations was evaluated. All signs of inflammation such as increase in spleen weight and granuloma formation in the liver were linked to CD4^+^ T cells. Our work reveals new mechanisms by which MAP induces inflammatory responses dependent on T-cell activity and might ultimately contribute to a better understanding of the role of MAP in chronic inflammatory disorders providing the basis for further investigations.

## Supporting Information

Figure S1 **Complete experiments of the panels shown in** **Figure 4** **including all controls.** MAP infected and CD4^+^CD45RB^lo/int^ T cell reconstituted (Inf&Rec) *Rag2* ^−/−^ mice. Control of *Rag2* ^−/−^ mice reconstituted with CD4^+^CD45RB^hi^ T cells not infected with MAP. *Rag2* ^−/−^ mice infected with MAP and reconstituted with CD4^+^CD45RB^hi^ T cells. All controls are shown. Arrow heads point at the granulomatous structures. Adoptive transfer of CD8^+^ T cells after MAP infection did not lead to formation of granulomatous structures. Bars depict 100 µm. The data are representative from 4 mice per group and the experiments were carried out at least twice with similar results.
(JPG)

Table S1 **Primers for qRT-PCR.** Table S1 presents ordering informations about the Quiagen primers used for qRT-PCR.
(DOCX)

Table S2 **Infection status at sacrifice.** Analytical methods and MAP infection status at sacrifice for subgroups infected with MAP (IHC immunohistochemistry, n.a. not analysed, rec./inf. reconstituted and infected).
(DOCX)
