# Supplementary figures and images for: Systemic and Mucosal Immune Reactivity upon Mycobacterium avium ssp. paratuberculosis Infection in Mice
Source: PLoS One. 2014 Apr 11;9(4):e94624. doi: 10.1371/journal.pone.0094624 (PMC3984212; doi:10.1371/journal.pone.0094624)

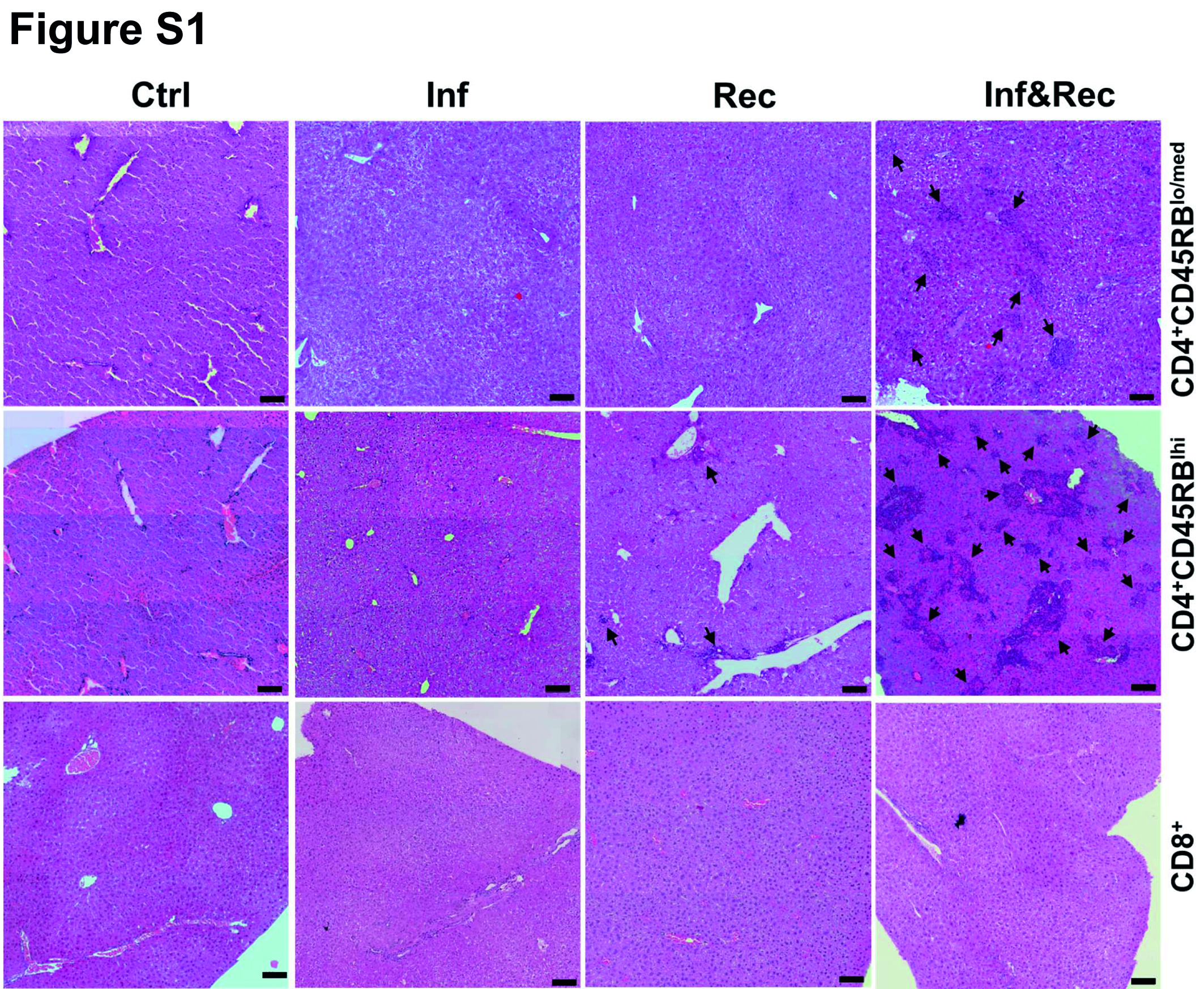

Supplement: Figure S1 — Complete experiments of the panels shown in Figure 4 including all controls. MAP infected and CD4+CD45RBlo/int T cell reconstituted (Inf&Rec) Rag2 −/− mice. Control of Rag2 −/− mice reconstituted with CD4+CD45RBhi T cells not infected with MAP. Rag2 −/− mice infected with MAP and reconstituted with CD4+CD45RBhi T cells. All controls are shown. Arrow heads point at the granulomatous structures. Adoptive transfer of CD8+ T cells after MAP infection did not lead to formation of granulomatous structures. Bars depict 100 µm. The data are representative from 4 mice per group and the experiments were carried out at least twice with similar results. (JPG) [file pone.0094624.s001.jpg]
