# Supplementary material for: Systemic and Mucosal Immune Reactivity upon Mycobacterium avium ssp. paratuberculosis Infection in Mice
Source: PLoS One. 2014 Apr 11;9(4):e94624. doi: 10.1371/journal.pone.0094624 (PMC3984212; doi:10.1371/journal.pone.0094624)
Supplement: Table S1 — Primers for qRT-PCR. Table S1 presents ordering informations about the Quiagen primers used for qRT-PCR. (DOCX) [file pone.0094624.s002.docx]

Table S1: Primers for qRT-PCR

Gene Cat. No.

r18S QT01036875

MMP-9 QT00108815

MMP-13 QT00111104

MMP-14 QT01064308

TIMP-1 QT00996282

TIMP-2 QT00138558

TLR-2 QT00129752

TLR-3 QT00122983

TLR-6 QT01078658

TLR-9 QT01748901

IL-1β QT01048355

TNF-α QT00104006)
