# Supplementary material for: Systemic and Mucosal Immune Reactivity upon Mycobacterium avium ssp. paratuberculosis Infection in Mice
Source: PLoS One. 2014 Apr 11;9(4):e94624. doi: 10.1371/journal.pone.0094624 (PMC3984212; doi:10.1371/journal.pone.0094624)
Supplement: Table S2 — Infection status at sacrifice. Analytical methods and MAP infection status at sacrifice for subgroups infected with MAP (IHC immunohistochemistry, n.a. not analysed, rec./inf. reconstituted and infected). (DOCX) [file pone.0094624.s003.docx]

**Table S2: Infection status at sacrifice**

| **reconstitution experiment** | **infection group** | **Liver (PCR/plating)** | **Granuloma (histology)** | **Colon (IHC)** | **MAP infection at least in one organ** |
| --- | --- | --- | --- | --- | --- |
| **CD4^+^CD45RB^lo/int^** | infected | 3/4 | 0/4 | 1/4 | 4/4 |
|  | rec./inf. | 1/4 | 1/4 | 4/4 | 4/4 |
| **CD4^+^CD45RB^hi^** | infected | 4/4 | 0/4 | 2/4 | 4/4 |
|  | rec./inf. | 4/4 | 4/4 | 4/4 | 4/4 |
| **CD8^+^** | infected | 2/4 | 0/4 | n.a. | 2/4 |
|  | rec./inf. | 4/4 | 1/4 | n.a. | 4/4 |

Table S2: Analytical methods and MAP infection status at sacrifice for subgroups infected with MAP (IHC immunohistochemistry, n.a. not analysed, rec./inf. reconstituted and infected).
